# Meckel–Gruber Syndrome: An Update on Diagnosis, Clinical Management, and Research Advances

**DOI:** 10.3389/fped.2017.00244

**Published:** 2017-11-20

**Authors:** Verity Hartill, Katarzyna Szymanska, Saghira Malik Sharif, Gabrielle Wheway, Colin A. Johnson

**Affiliations:** ^1^Department of Clinical Genetics, Yorkshire Regional Genetics Service, Leeds Teaching Hospitals NHS Trust, Leeds, United Kingdom; ^2^Leeds Institute of Biomedical and Clinical Sciences, University of Leeds, Leeds, United Kingdom; ^3^Faculty of Health and Applied Sciences, Department of Applied Sciences, UWE Bristol, Bristol, United Kingdom

**Keywords:** Meckel–Gruber syndrome, renal cystic dysplasia, oligohydramnios, primary cilium, transition zone, Shh signaling

## Abstract

Meckel–Gruber syndrome (MKS) is a lethal autosomal recessive congenital anomaly syndrome caused by mutations in genes encoding proteins that are structural or functional components of the primary cilium. Conditions that are caused by mutations in ciliary genes are collectively termed the ciliopathies, and MKS represents the most severe condition in this group of disorders. The primary cilium is a microtubule-based organelle, projecting from the apical surface of vertebrate cells. It acts as an “antenna” that receives and transduces chemosensory and mechanosensory signals, but also regulates diverse signaling pathways, such as Wnt and Shh, that have important roles during embryonic development. Most MKS proteins localize to a distinct ciliary compartment called the transition zone (TZ) that regulates the trafficking of cargo proteins or lipids. In this review, we provide an up-to-date summary of MKS clinical features, molecular genetics, and clinical diagnosis. MKS has a highly variable phenotype, extreme genetic heterogeneity, and displays allelism with other related ciliopathies such as Joubert syndrome, presenting significant challenges to diagnosis. Recent advances in genetic technology, with the widespread use of multi-gene panels for molecular testing, have significantly improved diagnosis, genetic counseling, and the clinical management of MKS families. These include the description of some limited genotype–phenotype correlations. We discuss recent insights into the molecular basis of disease in MKS, since the functions of some of the relevant ciliary proteins have now been determined. A common molecular etiology appears to be disruption of ciliary TZ structure and function, affecting essential developmental signaling and the regulation of secondary messengers.

## Clinical Features

Meckel–Gruber syndrome (MKS, OMIM number #249000), sometimes simply termed Meckel syndrome, was first described by Johann Friedrich Meckel in 1822. Meckel noted two siblings who died soon after birth with typical features that included occipital encephalocele, polycystic kidneys, and polydactyly (1). Gruber described the same condition later, naming it dysencephalia splanchnocystica (2). In the 1960s, the condition was further delineated by Opitz and Howe (3) with subsequent refinement of the diagnostic criteria (4–7).

**Figure 1:**
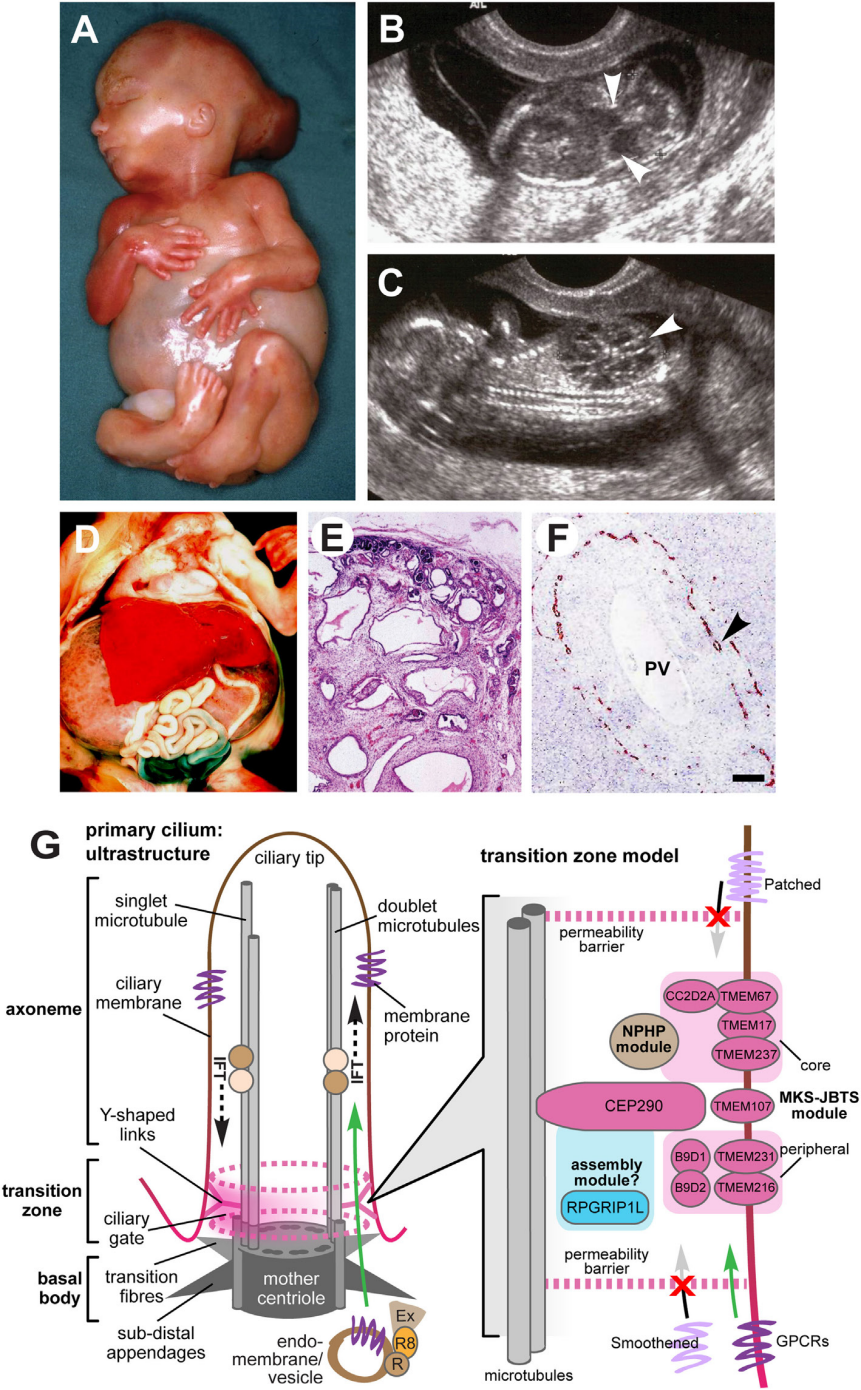
Clinical features of Meckel–Gruber syndrome (MKS) and schematic of primary cilia structure. **(A)** Typical external features for a fetus with MKS at gestation age 16/40, showing typical clinical features comprising occipital encephalocele, massive flank masses due to bilateral renal cystic dysplasia, postaxial hexadactyly of all limbs, and a typical Potter’s sequence facies with a slanting forehead and flattened nose. **(B,C)** Ultrasound findings at 14+/40 weeks of gestation for MKS showing **(B)** encephalocele (arrowheads), and **(C)** large cystic kidneys (arrowhead). **(D)** Massive swelling of the abdomen of a fetus at gestation age 18+/40 with MKS due to grossly enlarged, cystic kidneys. **(E)** Hematoxylin-eosin staining of MKS fetal kidney at gestation age 18+/40 showing cystic dysplasia, comprising large, fluid-filled cysts, small cysts, and cystic swelling of the proximal tubules and glomeruli, with the absence of normal renal parenchyma. **(F)** Immunohistochemical staining of MKS fetal liver at gestation age 18+/40 for cytokeratin-19, showing the retention of embryonic bile duct structures (the ductal plate malformation) without the formation of patent bile ducts (arrowhead). PV, hepatic portal vein. **(G)** Left: simplified schematic of cilium ultrastructure (individual components are not to scale). For the purposes of clarity, the ciliary axoneme is represented by two doublets of microtubules (the A- and B-tubules; gray rods), and the nine-fold symmetry in the structure is indicated by dark gray ovals in the mother centriole. The axoneme is bound by ciliary membrane (red/brown line and shading). The ciliary transition zone (TZ) is characterized by Y-shaped links (pink) that mediate interactions with the ciliary membrane. The permeability barrier called the “ciliary gate” is indicated by the dashed pink ovals and pink shading, and is thought to consist of transition fibers and TZ proteins. Ciliary cargo proteins (purple) are trafficking by vesicular transport mediated by coordinated interactions between the Rab family of small GTPases (RAB11 and RAB8; R8), the RAB8 activator protein Rabin8 (R), and the exocyst protein complex (Ex) at the ciliary base. The exocyst tethers vesicles to the periciliary membrane, facilitating transport through the ciliary gate (green arrow), followed by transport within the cilium mediated by intraflagellar transport (IFT; dashed black arrows). Right: schematic of a TZ model, showing the possible location of TZ proteins mutated in MKS and related ciliopathies (pink ovals) forming the “MKS-JBTS module,” RPGRIP1L (blue oval) which is thought to be a component of a ciliary assembly module, and a third functional module (brown) that is associated with nephronophthisis (NPHP) proteins. The selective transport of cargo transmembrane proteins (lilac or purple) is indicated by arrows. The proteins include G-protein-coupled receptors (GPCRs), and effectors of the Shh signaling pathway (Smoothened and Patched). Images **(A–C)** are used by kind permission of Dr. Riitta Salonen (Rinnekoti Foundation, Helsinki, Finland) from the Robert J. Gorlin Slide Collection. JBTS, Joubert syndrome.

Meckel–Gruber syndrome is a lethal developmental syndrome characterized by posterior fossa abnormalities (most frequently occipital encephalocele) (Figures 1A,B), bilateral enlarged cystic kidneys (Figures 1C–E), and hepatic developmental defects that include ductal plate malformation associated with hepatic fibrosis and cysts (Figure 1F) (8). A common additional feature is postaxial polydactyly, usually affecting both hands and feet (Figure 1A), which is seen in 70–80% of cases (5–7, 9). Other occasional features, seen in 25–40% of fetuses include bowing and shortening of the long bones, abnormalities of the male genitalia, microcephaly or anencephaly, cleft lip and palate, and other craniofacial abnormalities, congenital heart defects, and pulmonary hypoplasia (5, 6, 8, 9). Rare features (seen in less than 20% of cases) include cystic dysplasia of the lungs or thyroid, retinal colobomata, and *situs* defects (8–14). Central nervous system (CNS) defects are considered to be obligate features of MKS but appear to have a variable presentation, varying between total craniorachischisis, partial defects of the corpus callosum, Dandy–Walker malformation, and most frequently occipital encephalocele (15).

Renal cystic dysplasia is the most characteristic feature of MKS, but differs from those typical of polycystic kidney disease. The degree of cyst formation varies between individuals with MKS, but the kidneys will often be grossly enlarged, causing massive swelling of the abdomen (Figures 1A,D). Large, fluid-filled cysts are visible by eye in most affected individuals. However, in other small cysts, cystic changes of the proximal tubules and the absence of normal renal parenchyma are visible by microscopic investigation (Figure 1E). Cysts develop first in the glomeruli in the cortex, and cystogenesis progresses along the tubules and collecting ducts in the medulla. Abnormal fetal renal function is a frequent cause of oligohydramnios or anhydramnios, a common complication of an MKS pregnancy (7, 9). Potter’s sequence is frequent (pulmonary hypoplasia, growth restriction, club feet, and contractures) with a distinctive facies (comprising slanting forehead, flattened nose, and low-set ears) (Figure 1A), and is secondary to oligohydramnios or anhydramnios during pregnancy. In addition to cystic kidney dysplasia, hepatic involvement is an obligate feature of MKS (6). The typical histology shows bile duct proliferation and dilation (Figure 1F), associated with excess collagenous connective tissues (8), which are thought to arise from the incomplete development of the hepatic biliary system (16, 17).

## Genetics and Allelism

Autosomal recessive (AR) inheritance for MKS is confirmed by equal occurrence in males and females, concordance in monozygotic twins, examples of affected siblings, and, in some cases, parental consanguinity. If the condition has occurred in previous pregnancies then the recurrence risk is 25%. The worldwide incidence of MKS has been estimated at 1 in 135,000 live births (18), but higher incidences of MKS are observed in endogamous populations, such as Gujarati Indians (19), Tatars (20), Hutterites (21), and in Finland (22) where 1 in every 9,000 live births is affected. MKS incidence is also higher in consanguineous populations, such as Kuwaiti Bedouin tribes (1 in 3,530 births) (23) and populations in Saudi Arabia (1 in 3,500 births) (24).

Meckel–Gruber syndrome has extreme genetic heterogeneity and displays allelism with other ciliopathies such as Joubert syndrome (JBTS), COACH syndrome (cerebellar vermis hypo/aplasia, oligophrenia, congenital ataxia, ocular coloboma, and hepatic fibrosis), oro-facio-digital syndrome (OFD), nephronophthisis (NPHP), and Bardet–Biedl syndrome (BBS). To date, mutations in 14 genes (including *TXNDC15*) are identified as causative for MKS (Table 1). Mutations in a further three genes, specifically *C5orf42* (25), *CSPP1* (26), and *CEP55* (27), are implicated on the basis of pathogenic private mutations in individual families with either MKS or MKS-like phenotypes (Table 1). Two other genes, comprising *SEC8* (also known as *EXOC4*) (25) and *EXOC3L2* (28), have private mutations as a cause of probable MKS phenotypes. In total, mutations in these genes appear to explain only 50–60% of MKS cases (28, 29).

**Table 1:** Summary of known MKS loci and identified genes.

Locus	Gene	Entrez gene ID	Aliases	Location	Founder mutation(s)	Reference
MKS1	*MKS1*	54903	MKS, BBS13, JBTS28	17q22	Finnish c.1408-35_1408-7del29	(30)
MKS2	*TMEM216*	51259	JBTS2, CORS2	11q12.2	Ashkenazi c.218G > T (p.R73L)	(31, 32)
MKS3	*TMEM67*	91147	JBTS6, NPHP11, MECKELIN	8q22.1	Pakistani c.1575 + 1G > A	(33)
MKS4	*CEP290*	80184	KIAA0373, 3H11AG, JBTS5, SLSN6, LCA10, BBS14, NPHP6	12q21.32		(34)
MKS5	*RPGRIP1L*	23322	KIAA1005, JBTS7, CORS3, FTM, NPHP8	16q12.2	European c.1843A > C (p.T625P)	(35, 36)
MKS6	*CC2D2A*	57545	KIAA1345, JBTS9	4p15.32	Finnish c.1762C > T (p.?)	(37)
MKS7	*NPHP3*	27031	SLSN3, NPHP3, NPH3, RHPD1	3q22.1		(38)
MKS8	*TCTN2*	79867	C12orf38, TECT2, JBTS24	12q24.31		(39)
MKS9	*B9D1*	27077	MKSR1, JBTS27	17p11.2		(40)
MKS10	*B9D2*	80776	MKSR2	19q13.2		(41)
MKS11	*TMEM231*	79583	JBTS20	16q23.1		(42)
NA	*C5orf42*	65250	OFD6, JBTS17	5p13.2		(25)
NA	*CSPP1*	79848	JBTS21, CSPP	8q13.1-q13.2		(26)
MKS12	*KIF14*	9928	KIAA0042	1q32.1		(43)
MKS13	*TMEM107*	84314	JBTS29, PRO1268	17p13.1		(44, 45)
NA	*TXNDC15*	79770	C5orf14, UNQ335	5q31.1		(28)
NA	*CEP55*	55165	CT111, URCC6, C10orf3	10q23.33		(27)

*Assigned locus names are listed, with unassigned ones indicated by NA. Alias names of genes or loci summarize the alternative gene symbols, names, and any reported allelism with other ciliopathies (indicated by the gene symbol or locus name). Any reported founder mutations in specific populations are also listed, in addition to the original gene discovery paper or papers*.

*CORS, cerebellar–ocular–renal syndrome; JBTS, Joubert syndrome; MKS, Meckel–Gruber syndrome; NPHP, nephronophthisis; OFD, oro-facio-digital syndrome; SLSN, Senior-Løken syndrome*.

Mutations in *TMEM67, RPGRIP1L*, and *TMEM216* can cause both JBTS and MKS, demonstrating allelism between these conditions (31, 35, 36, 46) (Table 1). Biallelic mutations in the known MKS genes can also cause other related and allelic ciliopathies. Most notably, mutations in *CEP290* (*MKS4*) can also occur in patients with NPHP (47), Senior-Løken syndrome (SLSN), JBTS (34), BBS (48), COACH syndrome (49), and Leber congenital amaurosis (50). The variable expressivity of the MKS phenotype can complicate the diagnosis of the condition, since this can extend to intra-familial variation for individuals that carry the same pathogenic mutation within families (31), even between monozygotic twins (7). The effect of non-Mendelian inheritance modes or the influence of genetic modifier alleles has frequently been proposed to explain some of this variation. However, in a cohort of JBTS patients who shared causal alleles but had discordant phenotypic features, digenic, or oligogenic inheritance modes were largely excluded as disease mechanisms (51). A second recent study suggests that ciliopathy phenotypes, including those for MKS, are allele-specific and that stochastic effects have a more important role on phenotypic variability than modifier alleles (28).

## Genotype–Phenotype Correlations

In recent years, the identification of pathogenic alleles specific to MKS, limited genotype–phenotype correlations and founder mutations for particular ethnic groups (Table 1) have facilitated the rapid and accurate genetic diagnosis of this condition. Mutations in *MKS1* cause ca. 7% of all MKS cases and ca. 70% cases in Finland. Most Finnish MKS patients share the common “Finn major” mutation (*MKS1* IVS15-7_35del; Table 1), which is thought to have arisen in a founder of the Finnish population (52). Skeletal involvement including shortening and bowing of the long bones, polydactyly, and occipital encephalocele are frequently seen in patients with the Finn major mutation (18). More generally, *MKS1* mutations are associated with higher rates of polydactyly and occipital encephalocele, bone dysplasia, cleft palate, and *situs* defects (52). All *MKS1* mutations reported in MKS patients are null mutations, but a hypomorphic mutation has been reported in an individual with BBS (48), and recently in individuals with JBTS (53, 54).

*TMEM67* mutations are estimated to cause 16% of MKS cases (55), making them the most frequent cause of MKS. Both CNS malformations and polydactyly are less frequent in *TMEM67*-mutated individuals than those with *MKS1* mutations (56). *TMEM67* mutations also account for 57–83% (49, 55, 57) of the JBTS variant phenotype of COACH syndrome, demonstrating a strong genotype–phenotype correlation of *TMEM67* mutations with hepatic developmental defects or coloboma (49, 58). This correlation indicates that JBTS patients with hepatic involvement are prioritized for *TMEM67* mutation testing (57). Other genotype–phenotype correlations for MKS or JBTS have been identified by mutation type, or by the location of missense mutations within protein domains. For example, *CC2D2A* missense mutations are associated with JBTS whereas MKS is caused by null alleles (59). Missense mutations in exons 8–15 of *TMEM67*, particularly in combination with a second truncating mutation, are also associated with MKS (29, 55). Homozygous truncating mutations in *RPGRIP1L* are associated with MKS, whereas missense mutations (either in the homozygous state or compound heterozygous with a truncating mutation) are prevalent for JBTS patients. MKS phenotypes associated with *RPGRIP1L* mutations tend to be severe, including anencephaly and shortening and bowing of the long bones, in addition to the classic triad (36).

## Diagnosis, Clinical Management, and Genetic Counseling

Meckel–Gruber syndrome is lethal *in utero* or immediately after birth, often due to pulmonary hypoplasia, although an unusual survivor has been described aged 28 months (60). MKS has a broad, multi-organ phenotype with considerable variation, but it is generally diagnosed by the presence of cystic kidney dysplasia (Figure 1C), in addition to at least one other hallmark feature of the disease. These comprise occipital encephalocele (Figure 1B), polydactyly (Figure 1A), or hepatic developmental abnormalities such as the ductal plate malformation (Figure 1F) (6, 7). The presence of abnormal intrahepatic bile ducts and cystic kidneys has been proposed to be invariant features of MKS and appears to be pathognomonic for the condition (6, 17). Salonen (6) has proposed that the minimal diagnostic criteria for MKS are CNS malformation, bilateral multicystic kidneys, and hepatic fibrotic changes.

Transabdominal ultrasonography, performed at 10–14 weeks gestation, has been shown to successfully detect several of the fetal anomalies associated with MKS, including polycystic kidneys (from 9 weeks gestation), occipital encephalocele (from 13 weeks), and polydactyly (from 11 weeks), in both high-risk and low-risk pregnancies (Figure 1B) (60–64). Visualization can be compromised by oligohydramnios (65), but this is less problematic when performed in the first trimester of pregnancy (62). Further investigation of anomalies is possible by transvaginal scanning. Enlargement of the fetal trunk can give an early indication of multicystic renal dysplasia (66), in addition to unusually heterogeneous corticomedullary differentiation, reduced echogenicity of the medulla, increased echogenicity of the cortex, and the visualization of small medullary cysts (Figure 1C) (67). The fetal bladder can also be visualized by ultrasonography from 11 weeks, and the absence of a visible fetal bladder is often indicative of renal dysfunction.

Fetal MRI is an alternative if ultrasonography findings are inconclusive or lack of amniotic fluid prevents clear imaging. MRI offers better soft-tissue resolution than ultrasonography, and can provide clearer images of intracranial structures to enable an accurate diagnosis of CNS malformations, but is rarely performed before 18 weeks gestation. Fetal movement and maternal aortic pulsation do not preclude a successful diagnosis of MKS using MRI (68), since imaging artifacts caused by fetal movement can be minimized by a fetal neuromuscular blockade (69) or general anesthesia of the mother. Transabdominal or vaginal endoscopy in the first trimester of pregnancy allows diagnosis of MKS by visualization of the surface anatomy of the embryo. Fetoscopy enables the direct observation of polydactyly and occipital encephalocele from 11 weeks gestational age (70). Prenatal diagnosis is also possible by using a combination of these imaging techniques, α-fetoprotein testing of amniotic fluid, and DNA testing of fetus and parents. For example, elevated levels of maternal α-fetoprotein during antenatal screening may be associated with MKS (61).

Definitive diagnosis is often possible by using DNA testing to screen for mutations in the known MKS genes. Molecular diagnostic strategies include mutation screening of individual genes or targeted clonal sequencing of multi-gene panels. Single gene testing has low diagnostic sensitivity because of the absence of clear genotype–phenotype correlations for MKS. However, once a pathogenic variant in a family has been identified, then prenatal genetic diagnosis by chorionic villus sampling can be prioritized for at-risk pregnancies. Multi-gene panels can include MKS genes, and other ciliopathy genes of interest, that can be tested by sequence analysis and deletion/duplication analysis (71). However, their diagnostic sensitivity can vary between laboratories because panels can include genes that are unrelated to MKS. Referring clinicians should therefore decide which multi-gene panel has the best balance of sensitivity and affordability. If testing by a multi-gene panel does not confirm a clinical diagnosis of MKS, then more comprehensive testing by either whole-exome sequencing or whole-genome sequencing (WES or WGS) should be considered for obligate carriers. However, these tests may support or suggest unexpected diagnoses, in view of the broad range of possible differential diagnoses for MKS. These are extensive and include Smith–Lemli–Opitz syndrome, trisomy 13, hydrolethalus syndrome, and other related ciliopathies that include AR polycystic kidney disease, BBS, JBTS, and OFD. In particular, the prenatal features of BBS (polydactyly, renal defects, hepatic anomalies, genital hypoplasia, and heart malformations) can lead to a misdiagnosis of MKS (72). The extensive range of differential diagnoses and the broad allelism between different ciliopathies highlights the clinical need for accurate molecular diagnosis and the refinement of genotype–phenotype correlations (71, 73).

Since MKS has an AR inheritance pattern, couples with a previously affected child should have the opportunity for genetic counseling with a medical professional in order to discuss the nature, inheritance, and implications of an MKS diagnosis. The possibility of prenatal testing needs appropriate counseling of parents if it is being considered for the purpose of pregnancy termination in addition to early diagnosis. Individual opinions regarding prenatal testing and pregnancy termination may depend on a number of factors such as religious and cultural beliefs, and previous experiences of the medical condition (74, 75). An accurate perception of their genetic risk, a clear impact of genetic disorder and previous experience of having an affected child can help parents to make an informed decision (76). It is therefore appropriate to discuss these issues at an early stage to ensure that families can make informed medical and personal decisions.

With consanguineous MKS families, geneticists will encourage disclosure to at-risk relatives. The fact that these individuals often do not seek carrier testing suggests that many families are not sharing this information. In families with an AR disorder who practice consanguinity, the concern that their children’s carrier status would affect their marriage prospects can contribute to non-disclosure of this important information to their wider family (76–78). Furthermore, there is a widespread assumption among healthcare professionals that Muslim patients will not accept termination of pregnancy (79). However, under a *fatwā* (an Islamic legal pronouncement), termination of pregnancy is permitted before “ensoulment” at 120 days in cases of severe disability such as MKS, or where there is a risk to the mother’s life (75, 80), but knowledge of the *fatwā* is limited among Muslim populations (75, 81).

## Insights into Molecular Pathomechanisms

The primary cilium is a microtubule-based organelle, projecting from the apical surface of vertebrate cells. It acts as an “antenna” that receives and transduces chemosensory and mechanosensory signals (Figure 1G). Primary cilia are thought to be hubs of developmental signaling that regulate diverse pathways such as Wnt and Shh that have essential roles during embryonic development. In particular, mutations MKS genes cause defective Shh signaling in several mouse models of ciliopathies (82–85). Primary cilia have a complex architecture that defines the compartmentalization of ciliary proteins, and many genes that are mutated in MKS encode proteins that localize to the ciliary TZ. This is a distinct molecular compartment located at proximal regions of cilia (86, 87) that has been defined by genetic and biochemical approaches to consist of protein complexes forming the so-called “MKS-JBTS module” (Figure 1G). The TZ connects the microtubules of the ciliary axoneme to the plasma and ciliary membranes, and it has been suggested to both limit and assist the regulated trafficking of cargo proteins and lipids into and out of the cilium (88). The TZ is therefore thought to modulate the composition of essential ciliary compartments such as the ciliary membrane, axoneme, and associated proteins (88). A recent study demonstrated that the disruption of ciliary TZ architecture causes JBTS (89), but how this affects ciliary trafficking and causes the MKS or other ciliopathy phenotypes remains a research question of paramount importance.

Genetic, biochemical, and proteomic approaches have delineated the networks of protein-protein interactions that underlie the functional “MKS-JBTS module” at the TZ, as well providing insights into the structural basis of selective permeability at this ciliary compartment (90, 91). For example, several small tetraspanin-like transmembrane proteins (TMEMs) that are mutated as a cause of MKS and other ciliopathies (TMEM216, TMEM231, and TMEM107) appear to localize to the specialized ciliary membrane at the TZ. Other transmembrane proteins at this compartment include the Tectonic proteins (TCTN1, TCTN2, and TCTN3) and the Frizzled-like receptor TMEM67. In turn, the TMEM and Tectonic transmembrane proteins are likely to mediate interactions with and modulate the function of membrane-targeting proteins such as RPGRIP1L and CC2D2A that contain C2 domains (90, 91). TCTN1, for example, forms a biochemical complex at the TZ with CC2D2A, other ciliary transmembrane proteins (TCTN2, TMEM216, and TMEM67), and other known ciliopathy proteins (MKS1, CEP290, and B9D1) (92). The role of CEP290 and the B9 domain-containing proteins B9D1 (MKS9) and B9D2 (MKS10) at the TZ is undefined, but by analogy with the C2 domain-containing proteins, they are likely to mediate protein–protein interactions through their coiled-coil or B9 domains. One plausible model suggests that these proteins link TMEMs with either vesicular cargo at the TZ during the process of ciliogenesis, or then subsequently during the ciliary trafficking of cargo proteins through the TZ (Figure 1G). However, functional work on AHI1/jouberin, a ciliary TZ protein that contains WD40 and Src Homology 3 protein interaction domains, tends to support a general role for TZ proteins during vesicular trafficking because AHI1/jouberin interacts with RAB8A (93). RAB8A is an important small GTP/GDP-binding protein that is essential for vesicular protein trafficking from the endoplasmic reticulum to the Golgi apparatus and plasma membrane (93). Interestingly, AHI/jouberin also directly interacts and sequestrates β-catenin (a downstream effector of canonical Wnt signaling) at the cilium (94, 95) and it therefore has functions that are additional and distinct to those of CEP290.

Several recent studies have provided mechanistic insights into how the disruption of selective permeability at the TZ results in the incorrect trafficking of proteins at the cilium, with a focus on the trafficking of enzymes, receptors, and other transmembrane proteins involved in intracellular signaling. Although two TMEMs, TMEM17 and TMEM231, localize to the ciliary TZ, other TZ proteins (CC2D2A and B9D1) are required for TMEM231 localization to the TZ (82). In turn, these functional interactions in the TZ regulated the trafficking of ciliary G-protein-coupled receptors (GPCRs; somatostatin and serotonin receptors SSTR3 and HTR6) into the ciliary membrane, and both B9D2 and TMEM231 mutations caused defective ciliogenesis and Shh signaling in mice (82). In a separate study, loss of TCTN1 did not affect ciliogenesis, but nevertheless caused defective ciliary localizations of the transmembrane signaling proteins Smoothened and PKD2/polycystin-2, adenylyl cyclase 3 (ACIII; forming the secondary messenger cAMP), and ARL13B (a small Arf-family GTPase that localizes to the ciliary membrane) (92). Furthermore, the normal trafficking and localization of Smoothened at both the TZ and cilium is lost as a consequence of JBTS-associated mutations in RPGRIP1L that disrupt ciliary TZ architecture (89). In turn, the disruption of ciliary structure and function prevent Smoothened from mediating correct developmental Shh signaling (89). Disrupted compartmentalization could also prevent the correct ciliary transport of KIF7, a kinesin motor-protein that controls Shh signaling by regulating the amounts of activator and repressor isoforms of GLI transcription factors (96, 97). GLI proteins mediate gene expression changes as a consequence of Shh signaling, and the regulatory roles of KIF7 appear to be both negative, by preventing the incorrect activation of GLI2 in the absence of Shh ligand, and positive, by preventing the processing of GLI3 into the repressor isoform.

## Future Perspectives

At the present time, the molecular cause for only 60% of cases can be explained by mutations in the known MKS genes (28, 29, 71). The entire series of causative genes for MKS, MKS-like, and related ciliopathy phenotypes are therefore incomplete, but the remaining genes will be either uncommon or carry private mutations that are likely to be represented by single families. However, the widespread use of targeted clonal sequencing techniques such as multi-gene panels and WGS now allows the affordable re-investigation of molecular causes in the known MKS genes for individuals that have previously been mutation negative. These studies are likely to identify potential pathogenic causes that include intronic mutations, copy number variations, and other genic variants in promoter sequences or *cis*-regulatory elements. Although this will improve diagnostic rates for MKS families, these efforts are complicated by both allelism and extreme phenotypic variability for MKS and related ciliopathies. The mechanistic basis of phenotypic variability in MKS still remains largely unclear, and it is undefined what the relative contributions are made by modifier alleles in other genes, in contrast to the general stochastic effects of variability in developmental signaling during embryogenesis. A future research challenge is to delineate the complex relationships between ciliary architecture and organization, and how these relate to and regulate ciliary function.

## Author Contributions

VH, KS, SS, GW, and CJ made substantial contributions to the writing, drafting, and revision of this manuscript. All authors approved the final published version of the manuscript.

## Conflict of Interest Statement

The authors declare that the research was conducted in the absence of any commercial or financial relationships that could be construed as a potential conflict of interest.

## References

[1] Meckel J Beschreibung zweier, durch sehr ähnliche Bildungsabweichungen entstellter Geschwister. Dtsch Arch Physiol (1882) 7:99–172.

[2] Gruber G Beitrage zur frage “gekoppelter” Miszbildungen (Akrocephalo-syndactylie und dysencephalia splanchnocystica). Beitr Pathol Anat (1934) 93:459–76.

[3] Opitz JM Howe JJ The Meckel syndrome (dysencephalia splanchnocystica, the Grüber syndrome). Birth Defects Orig Art Ser (1969) 2:167–79.

[4] Mecke S Passarge E Encephalocele, polycystic kidneys and polydactyly as an autosomal recessive trait simulating certain other disorders – Meckel syndrome. Ann Genet (1971) 14:97–103. 4997715

[5] Fraser FC Lytwyn A Spectrum of anomalies in the Meckel syndrome, or maybe there is a malformation syndrome with at least one constant anomaly. Am J Med Genet (1981) 9:67–73. 10.1002/ajmg.1320090112 7246621

[6] Salonen R The Meckel syndrome – clinicopathological findings in 67 patients. Am J Med Genet (1984) 18:671–89. 10.1002/ajmg.1320180414 6486167

[7] Hsia YE Bratu M Herbordt A Genetics of Meckel syndrome (dysencephalia-splanchnocystica). Pediatrics (1971) 48:237–47. 4997860

[8] Salonen R Paavola P. Meckel syndrome. J Med Genet (1998) 35:497–501. 10.1136/jmg.35.6.497 9643292 PMC1051345

[9] Majewski F Stoss H Goecke T Kemperdick H. Are bowing of long tubular bones and preaxial polydactyly signs of the Meckel syndrome? Hum Genet (1983) 65:125–33. 10.1007/BF00286648 6654326

[10] Lowry RB Hill RH Tischler B. Survival and spectrum of anomalies in the Meckel syndrome. Am J Med Genet (1983) 14:417–21. 10.1002/ajmg.1320140303 6859092

[11] Moerman P Verbeken E Fryns JP Goddeeris P Lauweryns JM. The Meckel syndrome. Pathological and cytogenetic observations in eight cases. Hum Genet (1982) 62:240–5. 10.1007/BF00333528 6132872

[12] Seppanen U Herva R. Roentgenologic features of the Meckel syndrome. Pediatr Radiol (1983) 13:329–31. 10.1007/BF01625959 6646886

[13] Rapola J Salonen R. Visceral anomalies in the Meckel syndrome. Teratology (1985) 31:193–201. 10.1002/tera.1420310204 3992488

[14] Paavola P Salonen R Weissenbach J Peltonen L. The locus for Meckel syndrome with multiple congenital anomalies maps to chromosome 17q21-q24. Nat Genet (1995) 11:213–5. 10.1038/ng1095-213 7550354

[15] Ahdab-Barmada M Claassen D. A distinctive triad of malformations of the central nervous system in the Meckel-Gruber syndrome. J Neuropathol Exp Neurol (1990) 49:610–20. 10.1097/00005072-199011000-00007 2230839

[16] Blankenberg TA Ruebner BH Ellis WG Bernstein J Dimmick JE. Pathology of renal and hepatic anomalies in Meckel syndrome. Am J Med Genet Suppl (1987) 3:395–410. 10.1002/ajmg.1320280546 3130875

[17] Sergi C Adam S Kahl P Otto HF. Study of the malformation of ductal plate of the liver in Meckel syndrome and review of other syndromes presenting with this anomaly. Pediatr Dev Pathol (2000) 3:568–83. 10.1007/s100240010104 11000335

[18] Auber B Burfeind P Herold S Schoner K Simson G Rauskolb R A disease causing deletion of 29 base pairs in intron 15 in the *MKS1* gene is highly associated with the campomelic variant of the Meckel-Gruber syndrome. Clin Genet (2007) 72:454–9. 10.1111/j.1399-0004.2007.00880.x 17935508

[19] Young ID Rickett AB Clarke M High-incidence of Meckels syndrome in Gujarati Indians. J Med Genet (1985) 22:301–4. 10.1136/jmg.22.4.301 4045959 PMC1049454

[20] Lurie IW Prytkov AN Meldere LV. Meckel syndrome in different populations. Am J Med Genet (1984) 18:661–9. 10.1002/ajmg.1320180413 6486166

[21] Schurig V Bowen P Harley F Schiff D. The Meckel syndrome in the Hutterites. Am J Med Genet (1980) 5:373–81. 10.1002/ajmg.1320050408 7395917

[22] Salonen R Norio R. The Meckel syndrome in Finland: epidemiologic and genetic aspects. Am J Med Genet (1984) 18:691–8. 10.1002/ajmg.1320180415 6486168

[23] Teebi AS Alsaleh QA Odeh H Meckel syndrome and neural-tube defects in Kuwait. J Med Genet (1992) 29:140 10.1136/jmg.29.2.140-a PMC1015870 1613765

[24] Teebi AS Teebi SA. Genetic diversity among the Arabs. Community Genet (2005) 8:21–6. 10.1159/000083333 15767750

[25] Shaheen R Faqeih E Alshammari MJ Swaid A Al-Gazali L Mardawi E Genomic analysis of Meckel-Gruber syndrome in Arabs reveals marked genetic heterogeneity and novel candidate genes. Eur J Hum Genet (2013) 21:762–8. 10.1038/ejhg.2012.254 23169490 PMC3722952

[26] Shaheen R Shamseldin HE Loucks CM Seidahmed MZ Ansari S Ibrahim Khalil M Mutations in CSPP1, encoding a core centrosomal protein, cause a range of ciliopathy phenotypes in humans. Am J Hum Genet (2014) 94:73–9. 10.1016/j.ajhg.2013.11.010 24360803 PMC3882732

[27] Bondeson ML Ericson K Gudmundsson S Ameur A Pontén F Wesström J A nonsense mutation in CEP55 defines a new locus for a Meckel-like syndrome, an autosomal recessive lethal fetal ciliopathy. Clin Genet (2017) 92(5):510–6. 10.1111/cge.13012 28295209

[28] Shaheen R Szymanska K Basu B Patel N Ewida N Faqeih E Characterizing the morbid genome of ciliopathies. Genome Biol (2016) 17:242. 10.1186/s13059-016-1099-5 27894351 PMC5126998

[29] Szymanska K Berry I Logan CV Cousins SR Lindsay H Jafri H Founder mutations and genotype-phenotype correlations in Meckel-Gruber syndrome and associated ciliopathies. Cilia (2012) 1:18. 10.1186/2046-2530-1-18 23351400 PMC3579735

[30] Kyttälä M Tallila J Salonen R Kopra O Kohlschmidt N Paavola-Sakki P MKS1, encoding a component of the flagellar apparatus basal body proteome, is mutated in Meckel syndrome. Nat Genet (2006) 38:155–7. 10.1038/ng1714 16415886

[31] Valente EM Logan CV Mougou-Zerelli S Lee JH Silhavy JL Brancati F Mutations in TMEM216 perturb ciliogenesis and cause Joubert, Meckel and related syndromes. Nat Genet (2010) 42:619–25. 10.1038/ng.594 20512146 PMC2894012

[32] Edvardson S Shaag A Zenvirt S Erlich Y Hannon GJ Shanske AL Joubert syndrome 2 (JBTS2) in Ashkenazi Jews is associated with a TMEM216 mutation. Am J Hum Genet (2010) 86:93–7. 10.1016/j.ajhg.2009.12.007 20036350 PMC2801745

[33] Smith UM Consugar M Tee LJ McKee BM Maina EN Whelan S The transmembrane protein meckelin (MKS3) is mutated in Meckel-Gruber syndrome and the *wpk* rat. Nat Genet (2006) 38:191–6. 10.1038/ng1713 16415887

[34] Sayer J Otto EA O’Toole JF Nurnberg G Kennedy MA Becker C The centrosomal protein nephrocystin-6 is mutated in Joubert syndrome and activates transcription factor ATF4. Nat Genet (2006) 38:674–81. 10.1038/ng1786 16682973

[35] Arts HH Doherty D van Beersum SE Parisi MA Letteboer SJ Gorden NT Mutations in the gene encoding the basal body protein RPGRIP1L, a nephrocystin-4 interactor, cause Joubert syndrome. Nat Genet (2007) 39:882–8. 10.1038/ng2069 17558407

[36] Delous M Baala L Salomon R Laclef C Vierkotten J Tory K The ciliary gene RPGRIP1L is mutated in cerebello-oculo-renal syndrome (Joubert syndrome type B) and Meckel syndrome. Nat Genet (2007) 39:875–81. 10.1038/ng2039 17558409

[37] Tallila J Jakkula E Peltonen L Salonen R Kestilä M. Identification of CC2D2A as a Meckel syndrome gene adds an important piece to the ciliopathy puzzle. Am J Hum Genet (2008) 82:1361–7. 10.1016/j.ajhg.2008.05.004 18513680 PMC2427307

[38] Bergmann C Fliegauf M Brüchle NO Frank V Olbrich H Kirschner J Loss of nephrocystin-3 function can cause embryonic lethality, Meckel-Gruber-like syndrome, situs inversus, and renal-hepatic-pancreatic dysplasia. Am J Hum Genet (2008) 82:959–70. 10.1016/j.ajhg.2008.02.017 18371931 PMC2427297

[39] Shaheen R Faqeih E Seidahmed MZ Sunker A Alali FE AlQahtani K A TCTN2 mutation defines a novel Meckel Gruber syndrome locus. Hum Mutat (2011) 32:573–8. 10.1002/humu.21507 21462283

[40] Hopp K Heyer CM Hommerding CJ Henke SA Sundsbak JL Patel S B9D1 is revealed as a novel Meckel syndrome (MKS) gene by targeted exon-enriched next-generation sequencing and deletion analysis. Hum Mol Genet (2011) 20:2524–34. 10.1093/hmg/ddr151 21493627 PMC3109998

[41] Dowdle WE Robinson JF Kneist A Sirerol-Piquer MS Frints SG Corbit KC Disruption of a ciliary B9 protein complex causes Meckel syndrome. Am J Hum Genet (2011) 89:94–110. 10.1016/j.ajhg.2011.06.003 21763481 PMC3135817

[42] Shaheen R Ansari S Mardawi EA Alshammari MJ Alkuraya FS. Mutations in TMEM231 cause Meckel-Gruber syndrome. J Med Genet (2013) 50:160–2. 10.1136/jmedgenet-2012-101431 23349226 PMC3585488

[43] Filges I Nosova E Bruder E Tercanli S Townsend K Gibson WT Exome sequencing identifies mutations in KIF14 as a novel cause of an autosomal recessive lethal fetal ciliopathy phenotype. Clin Genet (2014) 86:220–8. 10.1111/cge.12301 24128419

[44] Shaheen R Almoisheer A Faqeih E Babay Z Monies D Tassan N Identification of a novel MKS locus defined by TMEM107 mutation. Hum Mol Genet (2015) 24:5211–8. 10.1093/hmg/ddv242 26123494

[45] Lambacher NJ Bruel AL van Dam TJ Szymańska K Slaats GG Kuhns S TMEM107 recruits ciliopathy proteins to subdomains of the ciliary transition zone and causes Joubert syndrome. Nat Cell Biol (2016) 18:122–31. 10.1038/ncb3273 26595381 PMC5580800

[46] Baala L Romano S Khaddour R Saunier S Smith UM Audollent S The Meckel-Gruber syndrome gene, MKS3, is mutated in Joubert syndrome. Am J Hum Genet (2007) 80:186–94. 10.1086/510499 17160906 PMC1785313

[47] Chang B Khanna H Hawes N Jimeno D He S Lillo C In-frame deletion in a novel centrosomal/ciliary protein CEP290/NPHP6 perturbs its interaction with RPGR and results in early-onset retinal degeneration in the rd16 mouse. Hum Mol Genet (2006) 15:1847–57. 10.1093/hmg/ddl107 16632484 PMC1592550

[48] Leitch CC Zaghloul NA Davis EE Stoetzel C Diaz-Font A Rix S Hypomorphic mutations in syndromic encephalocele genes are associated with Bardet-Biedl syndrome. Nat Genet (2008) 40:443–8. 10.1038/ng.97 18327255

[49] Brancati F Iannicelli M Travaglini L Mazzotta A Bertini E Boltshauser E MKS3/TMEM67 mutations are a major cause of COACH syndrome, a Joubert syndrome related disorder with liver involvement. Hum Mutat (2009) 30:E432–42. 10.1002/humu.20924 19058225 PMC2635428

[50] Den Hollander AI Koenekoop RK Yzer S Lopez I Arends ML Voesenek KEJ Mutations in the CEP290 (NPHP6) gene are a frequent cause of Leber congenital amaurosis. Am J Hum Genet (2006) 79:556–61. 10.1086/507318 16909394 PMC1559533

[51] Phelps IG Dempsey JC Grout ME Isabella CR Tully HM Doherty D Interpreting the clinical significance of combined variants in multiple recessive disease genes: systematic investigation of Joubert syndrome yields little support for oligogenicity. Genet Med (2017). 10.1038/gim.2017.94 28771248 PMC5797514

[52] Khaddour R Smith U Baala L Martinovic J Clavering D Shaffiq R Spectrum of MKS1 and MKS3 mutations in Meckel syndrome: a genotype-phenotype correlation. Mutation in brief #960. Online. Hum Mutat (2007) 28:523–4. 10.1002/humu.9489 17397051

[53] Bader I Decker E Mayr JA Lunzer V Koch J Boltshauser E MKS1 mutations cause Joubert syndrome with agenesis of the corpus callosum. Eur J Med Genet (2016) 59:386–91. 10.1016/j.ejmg.2016.06.007 27377014

[54] Irfanullah F Khan S Ullah I Nasir A Meijer CA Laurense-Bik M Hypomorphic MKS1 mutation in a Pakistani family with mild Joubert syndrome and atypical features: expanding the phenotypic spectrum of MKS1-related ciliopathies. Am J Med Genet A (2016) 170:3289–93. 10.1002/ajmg.a.37934 27570071

[55] Iannicelli M Brancati F Mougou-Zerelli S Mazzotta A Thomas S Elkhartoufi N Novel TMEM67 mutations and genotype-phenotype correlates in meckelin-related ciliopathies. Hum Mutat (2010) 31:E1319–31. 10.1002/humu.21239 20232449 PMC2918781

[56] Consugar M Kubly V Lager D Hommerding C Wong W Bakker E Molecular diagnostics of Meckel-Gruber syndrome highlights phenotypic differences between MKS1 and MKS3. Hum Genet (2007) 121:591–9. 10.1007/s00439-007-0341-3 17377820

[57] Doherty D Parisi MA Finn LS Gunay-Aygun M Al-Mateen M Bates D Mutations in 3 genes (MKS3, CC2D2A and RPGRIP1L) cause COACH syndrome (Joubert syndrome with congenital hepatic fibrosis). J Med Genet (2010) 47:8–21. 10.1136/jmg.2009.067249 19574260 PMC3501959

[58] Otto EA Tory K Attanasio M Zhou W Chaki M Paruchuri Y Hypomorphic mutations in meckelin (MKS3/TMEM67) cause nephronophthisis with liver fibrosis (NPHP11). J Med Genet (2009) 46:663–70. 10.1136/jmg.2009.066613 19508969

[59] Mougou-Zerelli S Thomas S Szenker E Audollent S Elkhartoufi N Babarit C CC2D2A mutations in Meckel and Joubert syndromes indicate a genotype-phenotype correlation. Hum Mutat (2009) 30:1574–82. 10.1002/humu.21116 19777577 PMC2783384

[60] Ramadani HM Nasrat HA. Prenatal diagnosis of recurrent Meckel syndrome. Int J Gynaecol Obstet (1992) 39:327–32. 10.1016/0020-7292(92)90265-K 1361467

[61] Sepulveda W Sebire NJ Souka A Snijders RJM Nicolaides KH. Diagnosis of the Meckel-Gruber syndrome at eleven to fourteen weeks’ gestation. Am J Obstet Gynecol (1997) 176:316–9. 10.1016/S0002-9378(97)70491-5 9065174

[62] Braithwaite JM Economides DL. First-trimester diagnosis of Meckel-Gruber syndrome by transabdominal sonography in a low-risk case. Prenat Diagn (1995) 15:1168–70. 10.1002/pd.1970151215 8750299

[63] Pachi A Giancotti A Torcia F Deprosperi V Maggi E Meckel-Gruber syndrome – ultrasonographic diagnosis at 13 weeks gestational age in an at-risk case. Prenat Diagn (1989) 9:187–90. 10.1002/pd.1970090307 2652130

[64] Nizard J Bernard JP Ville Y. Fetal cystic malformations of the posterior fossa in the first trimester of pregnancy. Fetal Diagn Ther (2005) 20:146–51. 10.1159/000082440 15692211

[65] Verjaal M Meyer AH Beckerbloemkolk MJ Leschot NJ Weduwen JJD Gras J Oligohydramnios hampering prenatal-diagnosis of Meckel syndrome. Am J Med Genet (1980) 7:85–6. 10.1002/ajmg.1320070112 7211955

[66] Kaffe S Rose JS Godmilow L Walker BA Kerenyi T Beratis N Prenatal diagnosis of renal anomalies. Am J Med Genet (1977) 1:241–51. 10.1002/ajmg.1320010210 610432

[67] Ickowicz V Eurin D Maugey-Laulom B Didier F Garel C Gubler MC Meckel-Gruber syndrome: sonography and pathology. Ultrasound Obstet Gynecol (2006) 27:296–300. 10.1002/uog.2708 16450359

[68] Chao A Wong AM Hsueh C Chang YL Wang TH Integration of imaging and pathological studies in Meckel-Gruber syndrome. Prenat Diagn (2005) 25:267–8. 10.1002/pd.1111 15791677

[69] Williamson RA Weiner CP Yuh WTC Abuyousef MM Magnetic-resonance imaging of anomalous fetuses. Obstet Gynecol (1989) 73:952–6. 2657526 10.1097/00006250-198906000-00009

[70] Quintero RA Abuhamad A Hobbins JC Mahoney MJ Transabdominal thin-gauge embryofetoscopy – a technique for early prenatal diagnosis and its use in the diagnosis of a case of Meckel-Gruber syndrome. Am J Obstet Gynecol (1993) 168:1552–7. 10.1016/S0002-9378(11)90797-2 8498442

[71] Watson CM Crinnion LA Berry IR Harrison SM Lascelles C Antanaviciute A Enhanced diagnostic yield in Meckel-Gruber and Joubert syndrome through exome sequencing supplemented with split-read mapping. BMC Med Genet (2016) 17:1. 10.1186/s12881-015-0265-z 26729329 PMC4700600

[72] Karmous-Benailly H Martinovic J Gubler MC Sirot Y Clech L Ozilou C Antenatal presentation of Bardet-Biedl syndrome may mimic Meckel syndrome. Am J Hum Genet (2005) 76:493–504. 10.1086/428679 15666242 PMC1196400

[73] Chen CP. Meckel syndrome: genetics, perinatal findings, and differential diagnosis. Taiwan J Obstet Gynecol (2007) 46:9–14. 10.1016/S1028-4559(08)60100-X 17389183

[74] Ahmed S Green JM Hewison J. Attitudes towards prenatal diagnosis and termination of pregnancy for thalassaemia in pregnant Pakistani women in the North of England. Prenat Diagn (2006) 26:248–57. 10.1002/pd.1391 16475227

[75] Ahmed S Ahmed M Sharif SM Sheridan E Taylor GR. Attitudes towards prenatal testing and termination of pregnancy in British Pakistani parents and relatives of children with recessive conditions in the UK. Prenat Diagn (2012) 32:954–9. 10.1002/pd.3940 22806755

[76] Ahmed S Bryant LD Cole P. Midwives’ perceptions of their role as facilitators of informed choice in antenatal screening. Midwifery (2013) 29:745–50. 10.1016/j.midw.2012.07.006 22901497

[77] Middleton A Robson F Burnell L Ahmed M. Providing a transcultural genetic counseling service in the UK. J Genet Couns (2007) 16:567–82. 10.1007/s10897-007-9089-0 17492497

[78] Ali TS Gunilla K Gul R Asad N Johansson E Mogren I. Gender roles and their influence on life prospects for women in urban Karachi, Pakistan: a qualitative study. Glob Health Action (2011) 4:7448. 10.3402/gha.v4i0.7448 22065609 PMC3208374

[79] Modell B Harris R Lane B Khan M Darlison M Petrou M Informed choice in genetic screening for thalassaemia during pregnancy: audit from a national confidential inquiry. BMJ (2000) 320:337–41. 10.1136/bmj.320.7231.337 10657326 PMC27278

[80] Al-Matary A Ali J. Controversies and considerations regarding the termination of pregnancy for foetal anomalies in Islam. BMC Med Ethics (2014) 15:10. 10.1186/1472-6939-15-10 24499356 PMC3943453

[81] Jafri H Ahmed S Ahmed M Hewison J Raashid Y Sheridan E Islam and termination of pregnancy for genetic conditions in Pakistan: implications for Pakistani health care providers. Prenat Diagn (2012) 32:1218–20. 10.1002/pd.3987 23080051

[82] Chih B Liu P Chinn Y Chalouni C Komuves LG Hass PE A ciliopathy complex at the transition zone protects the cilia as a privileged membrane domain. Nat Cell Biol (2011) 14:61–72. 10.1038/ncb2410 22179047

[83] Cui C Chatterjee B Francis D Yu Q SanAgustin JT Francis R Disruption of Mks1 localization to the mother centriole causes cilia defects and developmental malformations in Meckel-Gruber syndrome. Dis Model Mech (2011) 4:43–56. 10.1242/dmm.006262 21045211 PMC3008963

[84] Weatherbee SD Niswander LA Anderson KV. A mouse model for Meckel syndrome reveals Mks1 is required for ciliogenesis and Hedgehog signaling. Hum Mol Genet (2009) 18:4565–75. 10.1093/hmg/ddp422 19776033 PMC2773271

[85] Abdelhamed ZA Wheway G Szymanska K Natarajan S Toomes C Inglehearn C Variable expressivity of ciliopathy neurological phenotypes that encompass Meckel-Gruber syndrome and Joubert syndrome is caused by complex de-regulated ciliogenesis, Shh and Wnt signalling defects. Hum Mol Genet (2013) 22:1358–72. 10.1093/hmg/dds546 23283079 PMC3596847

[86] Szymanska K Johnson CA. The transition zone: an essential functional compartment of cilia. Cilia (2012) 1:10. 10.1186/2046-2530-1-10 23352055 PMC3555838

[87] Reiter JF Blacque OE Leroux MR. The base of the cilium: roles for transition fibres and the transition zone in ciliary formation, maintenance and compartmentalization. EMBO Rep (2012) 13:608–18. 10.1038/embor.2012.73 22653444 PMC3388784

[88] Williams CL Li C Kida K Inglis PN Mohan S Semenec L MKS and NPHP modules cooperate to establish basal body/transition zone membrane associations and ciliary gate function during ciliogenesis. J Cell Biol (2011) 192:1023–41. 10.1083/jcb.201012116 21422230 PMC3063147

[89] Shi X Garcia G III Van De Weghe JC McGorty R Pazour GJ Doherty D Super-resolution microscopy reveals that disruption of ciliary transition-zone architecture causes Joubert syndrome. Nat Cell Biol (2017) 19(10):1178–88. 10.1038/ncb3599 28846093 PMC5695680

[90] Garcia-Gonzalo FR Reiter JF. Scoring a backstage pass: mechanisms of ciliogenesis and ciliary access. J Cell Biol (2012) 197:697–709. 10.1083/jcb.201111146 22689651 PMC3373398

[91] Blacque OE Sanders AA. Compartments within a compartment: what *C. elegans* can tell us about ciliary subdomain composition, biogenesis, function, and disease. Organogenesis (2014) 10:126–37. 10.4161/org.28830 24732235 PMC4049889

[92] Garcia-Gonzalo FR Corbit KC Sirerol-Piquer MS Ramaswami G Otto EA Noriega TR A transition zone complex regulates mammalian ciliogenesis and ciliary membrane composition. Nat Genet (2011) 43:776–84. 10.1038/ng.891 21725307 PMC3145011

[93] Hsiao YC Tong ZJ Westfall JE Ault JG Page-McCaw PS Ferland RJ. AHI1, whose human ortholog is mutated in Joubert syndrome, is required for Rab8a localization, ciliogenesis and vesicle trafficking. Hum Mol Genet (2009) 18:3926–41. 10.1093/hmg/ddp335 19625297 PMC2748898

[94] Lancaster MA Louie CM Silhavy JL Sintasath L Decambre M Nigam SK Impaired Wnt-beta-catenin signaling disrupts adult renal homeostasis and leads to cystic kidney ciliopathy. Nat Med (2009) 15:1046–54. 10.1038/nm.2010 19718039 PMC2895985

[95] Lancaster MA Schroth J Gleeson JG. Subcellular spatial regulation of canonical Wnt signalling at the primary cilium. Nat Cell Biol (2011) 13:700–7. 10.1038/ncb2259 21602792 PMC3107376

[96] Dafinger C Liebau MC Elsayed SM Hellenbroich Y Boltshauser E Korenke GC Mutations in KIF7 link Joubert syndrome with Sonic Hedgehog signaling and microtubule dynamics. J Clin Invest (2011) 121:2662–7. 10.1172/JCI43639 21633164 PMC3223820

[97] Putoux A Thomas S Coene KL Davis EE Alanay Y Ogur G KIF7 mutations cause fetal hydrolethalus and acrocallosal syndromes. Nat Genet (2011) 43:601–6. 10.1038/ng.826 21552264 PMC3674836

